# Non-invasive Raman Spectroscopy and Quantitative Real-Time PCR Distinguish Among Undifferentiated Human Mesenchymal Stem Cells and Redifferentiated Nucleus Pulposus Cells and Chondrocytes *In Vitro*

**DOI:** 10.2174/1874120701711010072

**Published:** 2017-07-31

**Authors:** Franziska Ehlicke, Natascha Köster, Denise Salzig, Peter Czermak

**Affiliations:** 1 Institute of Bioprocess Engineering and Pharmaceutical Technology, University of Applied Sciences Mittelhessen, Wiesenstr 14, 35390 Giessen, Germany; 2 Department of Chemical Engineering, Kansas State University, Manhattan, KS 66506, USA; 3 Faculty of Biology and Chemistry, Justus-Liebig-University of Giessen, Ludwigstr. 23, 35390 Giessen, Germany; 4 Fraunhofer Institute for Molecular Biology and Applied Ecology (IME), Project Group Bioresources, Winchesterstr. 3, 35394 Giessen, Germany; 5 Department Tissue Engineering and Regenerative Medicine, University Hospital Wuerzburg, Roentgenring 11, 97070 Wuerzburg, Germany

**Keywords:** Intervertebral disc regeneration, Mesenchymal stem cells, Nucleus pulposus cells, Chondrocytes, Characterization, Distinction, Cell markers, Raman spectroscopy

## Abstract

**Background:**

The most common cause of lower back pain is the pathological degeneration of the nucleus pulposus (NP). Promising NP regeneration strategies involving human mesenchymal stem cells (hMSCs) would require specific markers to confirm successful differentiation into the NP lineage and to distinguish the articular cartilage (AC).

**Objective:**

We sought specific NP mRNA markers that are upregulated in native NP cells but not in dedifferentiated NP cells, undifferentiated hMSCs or chondrocytes. We also considered the suitability of non-invasive Raman spectroscopy to distinguish among these classes of cells.

**Method:**

We used quantitative real-time PCR and Raman spectroscopy to analyse undifferentiated hMSCs in monolayers and embedded in hydrogels, and compared the results with dedifferentiated and redifferentiated human NP and AC cells.

**Results:**

The redifferentiation of NP cells induced the expression of annexin A3 (*ANXA3*), collagen type II (*COL2*) and proteoglycan mRNAs, whereas the redifferentiation of AC cells only induced proteoglycan expression. Redifferentiated NP cells expressed higher levels of *ANXA3*, *COL2*, paired box 1 (*PAX1*) and *OCT4* mRNA than redifferentiated AC cells. Redifferentiated NP cells and undifferentiated hMSC-TERT cells expressed similar amount of *OCT4* mRNA, indicating that only *ANXA3*, *COL2* and *PAX1* are promising markers for redifferentiated NP cells. Raman spectra clearly differed among the three cell types and highlighted their differentiation status.

**Conclusion:**

We recommend *ANXA3*, *COL2* and *PAX1* as markers to determine the success of hMSC-based differentiation to regenerate NP cells. Raman spectroscopy can be used to determine cell type and differentiation status especially in the context of clinical trials.

## 1 INTRODUCTION

Disorders of the intervertebral disc (IVD) are common in modern society, causing severe pain for patients and a high cost burden on national health systems [1, 2]. The IVD is a complex structure that can be separated into three distinct zones: the central nucleus pulposus (NP) surrounded circumferentially by the annulus fibrosus (AF) and cartilage end plates (CEPs) adjacent to the vertebral bodies. The three tissues differ in terms of the cell types they contain and the composition of the extracellular matrix (ECM). The CEPs are derived from the sclerotome and consist of chondrocytes and hyaline cartilage [3]. These structures supply nutrients to and remove waste from the entire IVD, although their capacity is limited by calcification. The AF is also derived from the sclerotome, but contains fibroblast-like and chondrocyte-like cells [4] embedded in an ECM consisting mainly of type-I collagen with small amounts of proteoglycan (PG) or type-II collagen [5]. In contrast, the NP is derived from the notochord and in humans consists of notochordal cells (NCs) until the end of the first life decade, and then chondrocyte-like NP cells during adolescence and adulthood [6]. The ECM produced by the NP cells is richly hydrated and contains abundant PGs (predominantly aggrecan) and type-II collagen. The gel-like NP together with the fibrous AF functions as a shock absorber and in healthy individuals enables the three-dimensional movement of the spine.

IVD degeneration may begin in the NP with changes in gene expression that reduce the proteoglycan content. The NP therefore becomes stiffer and more cartilaginous, and the cells cease to proliferate and eventually undergo apoptosis [7]. Due to its avascular nature and the disappearance of NCs during childhood, the NP in humans has an extremely low capacity for self-regeneration [8]. Treatment options for IVD degeneration are therefore almost entirely limited to the alleviation of pain [9, 10]. In contrast, therapeutic strategies focus on the regeneration of NP tissue and the restoration of its shock-absorbing function. Despite promising results *in vitro* and in preclinical studies, few clinical trials have been reported [11].

The success of NP regeneration requires detailed information concerning the developmental behaviour of the NP cells. The gene expression profile of NP cells has been compared to that of the articular cartilage (AC) and AF cells [12], but there is limited access to healthy human NP tissue so most studies are based on animal tissues [13-19]. These data cannot be mapped directly to human patients because gene expression profiles are species-dependent [14, 17]. Several human NP markers have been defined, including genes encoding forkhead box F1 (*FOXF1*), ovostatin (*OVOS*), haemoglobin beta chain (*HBB*), carbonic anhydrase XII (*CA12*), paired box 1 (*PAX1*), keratin 18 (*KRT18*), keratin 19 (*KRT19*) [20], and cadherin 2 (*CDH2*) [21]. In 2015, the Spine Research Interest Group recommended the stabilized expression of HIF-1α, GLUT-1, SHH, Brachyury (*T*), KRT18/19, CA12 and CD24, together with an aggrecan/collagen II ratio > 20 to define a healthy NP phenotype [22]. However, according to our knowledge, no studies have yet compared human NP cells with human mesenchymal stem cells (hMSCs), even though the latter are widely used as the raw material for NP regeneration attempts and it is therefore necessary to reliably distinguish between them [23-25]. Our own previous experiments [26] revealed that the reported NP-specific marker KRT19 [20, 21] is expressed more strongly in the cell line hMSC-TERT than in commercially available human NP cells (ScienCell, Carlsbad, CA, USA) indicating that current NP markers may not be sufficient to distinguish NP cells in all experimental contexts. The remaining NP markers should also be subjected to rigorous testing to ensure they can distinguish between NP cells and hMSCs.

Specific markers for human NP cells are necessary to develop cell therapy strategies and protocols. Nevertheless, the detection of such markers by quantitative real-time RT-PCR (qRT-PCR) or histological staining is destructive, preventing the therapeutic application of cells that have passed quality control. Therefore, non-invasive techniques such as Raman spectroscopy should be used for cell characterization. Raman spectroscopy is based on the inelastic scattering of monochromatic light at different wavelengths by different samples. These so-called Raman shifts (or wavenumbers [cm^-1^]) are assigned to molecular vibrations, therefore creating specific Raman spectra (biological fingerprints) for different sample compositions, such as cells at different stages of differentiation [27-29]. This approach has been used to analyse hMSCs and chondrocytes in monolayers or suspension [30], to compare spectral data for chondrocytes and ECM taken from the superficial and middle/deep zones of human cartilage slices [28], and to compare distinct zones of the growth plate of human foetal femur cartilage [31]. However, to the best of our knowledge, NP cells have never been analysed by Raman spectroscopy.

Here, we aimed to identify specific NP marker genes that are induced in native NP cells but not in dedifferentiated NP cells, undifferentiated hMSCs or chondrocytes. We also aimed to demonstrate the ability of Raman spectroscopy to distinguish among undifferentiated hMSCs, differentiated NP cells and chondrocytes. Because the redifferentiation of NP cells [32] and chondrocytes [33] as well as successful differentiation of hMSCs requires a three dimensional (3D) culture, we aimed to distinguish among the three cell types in 3D agarose hydrogels. Redifferentiated NP cells and chondrocytes were analysed 21 days after seeding in order to achieve phenotypes similar to the native population. Undifferentiated hMSC-TERT cells were analysed after 1 day. To ensure robust analysis, we also compared the Raman spectra of dedifferentiated and redifferentiated NP cells. We recommend primers and qRT-PCR conditions to identify putative markers for NP cells, chondrocytes and hMSCs.

## 2 MATERIALS AND METHOD

### 2.1 Monolayer Culture

Human mesenchymal stem cells with reverse telomerase transcriptase (hMSC-TERT) [34] were cultured in Eagle’s minimal essential medium (EMEM; PAA, Pasching, Austria) supplemented with 10% foetal bovine serum (FBS; PAA), 100 U/mL penicillin and 100 μg/mL streptomycin (both Biochrom, Berlin, Germany) as previously described [35]. Human NP cells (male, foetal, 20 weeks old) were obtained from ScienCell (Carlsbad, CA, USA) and expanded in Human Nucleus Pulposus Cell Medium containing 2% FBS and 1% growth supplement (ScienCell). Human chondrocytes (male, 65 years old) were obtained from Provitro (Berlin, Germany) and cultured in Chondrocyte Growth Medium containing 10% FBS (Provitro). All three cell types were cultured in different culture formats (25-300 cm^2^ flasks) in a humidified incubator at 37°C with a 5% CO_2_ atmosphere. Cells were passaged at 80% confluency and used during passages 69–75 (hMSC-TERT), 2-8 (NP cells) and 5-7 (chondrocytes). Cell morphology was analysed using a wide-field microscope.

### 2.2 Hydrogel Culture

The human NP cells and chondrocytes were redifferentiated in hydrogels comprising 2% (w/v) low-gelling-point agarose type VII (Sigma-Aldrich, Steinheim, Germany). We embedded 1.2 x 10^6^ cells in 300 μL liquid agarose in 48-well plates (the agarose was maintained below 40°C to protect the cells) and the solidified hydrogel was then transferred to 24-well plates and overlaid with 1.5 mL of the appropriate medium. The hMSC-TERT cells were cultivated for 1 day, whereas the NP cells and chondrocytes were cultured for 21 days with medium exchange every 3-4 days.

### 2.3 RNA Isolation

RNA was extracted from monolayer cells using the RNeasy Mini Kit (Qiagen, Hilden, Germany) according to the manufacturer’s recommendations. RNA was extracted from hydrogel-embedded cells by microhomogenization followed by the CTAB method as previously described [35]. The RNA yield was determined according to the Lambert-Beer law by measuring A_260_ and RNA purity was determined using the A_260_/A_280_ ratio (pure RNA has a ratio of 2.0). The RNA was stored at -20°C.

### 2.4 Reverse Transcription

For cDNA synthesis, 2 µg RNA was transcribed using the Precision nanoScript™ Reverse Transcription Kit including oligo-dT primer from Primerdesign Ltd (Southampton, United Kingdom) according to the manufacturer’s recommendations.

### 2.5 Quantitative Real-Time Polymerase Chain Reaction

The qRT-PCR experiments were carried out using the Precision^TM^ 2X qPCR Mastermix Kit with SYBRgreen^®^ detection according to the manufacturer’s protocol (Primerdesign Ltd) with eukaryotic translation initiation factor 4A2 (*EIF4A2*) as the reference gene, based on results obtained with the Human Genome Reference Gene Selection Kit (Primerdesign Ltd). The selected genes (Table **1**) were amplified using a Mastercycler^®^ ep *realplex* (Eppendorf AG, Hamburg, Germany). Relative gene expression levels were determined using the ΔΔCt method in REST2009 v2.0.13 (Qiagen and Prof. Dr. Michael Pfaffl, Technical University of Munich, Germany) including statistical data analysis (Web ref. 1).

### 2.6 Raman Spectroscopy

#### 2.6.1 Data Acquisition

Monolayer cells were trypsinized and resuspended in phosphate buffered saline (PBS) prior to Raman spectroscopy, whereas embedded cells were immersed in PBS to prevent dehydration. For data acquisition, a custom-built Raman spectrometer was connected to an Olympus IX71 fluorescence microscope (Olympus, Hamburg, Germany) and equipped with an 85-mW, 785-nm diode laser (TOPTICA Photonics, Gräfelfing, Germany), a blocking filter to separate the elastic Rayleigh scattering, and a spectrograph (Kaiser Optical Systems, Ann Arbor, MI, USA) with an iDus CCD camera (Andor Technology, Belfast, UK). Thirty individual cells were analysed per experiment with 100 s integration time. Data were acquired using Andor Solis software (Andor Technology) and Cell^B software (Olympus). A background spectrum was taken from each focal plane for subsequent data analysis.

#### 2.6.2 Data Analysis

Background spectrum subtraction, baseline correction and spike removal were carried out using OPUS v7 (Bruker Optik, Ettlingen, Germany). Spectra were cut into the range 600-1800 cm^-1^ and compared by principal component analysis (PCA) with The Unscrambler^®^ software v10.2 (CAMO Software AS, Oslo, Norway). Seven principal components (PCs) were calculated for each comparison using the nonlinear iterative partial least squares (NIPALS) algorithm. Score plots were created representing the two PCs achieving the best data separation. The PC loadings were analysed to determine which wavenumbers contributed most to the PC, and further PCA was carried out with spectral data within this specific wavenumber range.

## 3 RESULTS

### 3.1 Developmental Characteristics of Cells During Dedifferentiation and Redifferentiation

**Fig. (1):**
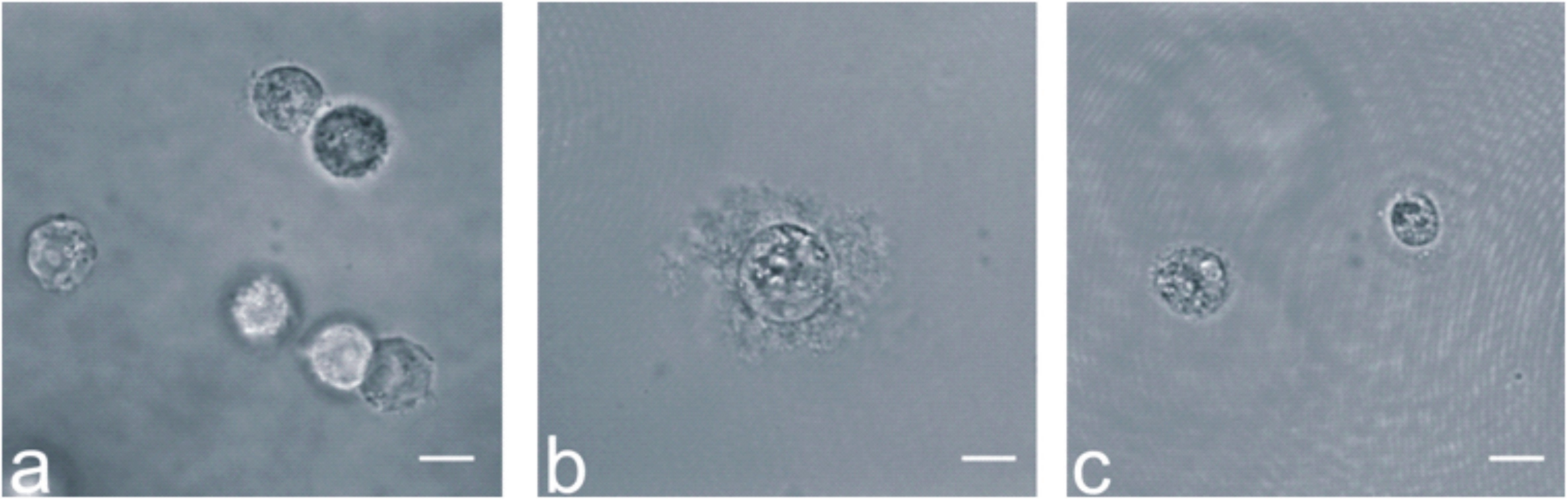
Wide-field images of in agarose-embedded cells: (**a**) hMSC-TERT cells after 1 day, (**b**) NP cells and (**c**) chondrocytes after 21 days in hydrogel culture. Scale bars = 10 µm.

The dedifferentiation of NP cells and chondrocytes was induced by expanding them in a monolayer, and redifferentiation was then promoted in a 3D culture environment by embedding them in hydrogels. We found that NP cells clearly showed evidence of ECM production after 21 days culture in agarose hydrogels, whereas chondrocytes produced only small amounts of ECM (Fig. **1**).

### 3.2 Gene Expression Profiles: Comparison of Cells in Monolayers and Hydrogels

The hMSC-TERT cells, NP cells and chondrocytes were expanded in monolayers and subsequently cultivated in 3D hydrogels for redifferentiation. The expression profiles for each cell type in the different culture setups were compared by measuring the abundance of mRNAs representing selected genes (Table **1**). The comparative expression levels for each cell type in hydrogels and monolayer cultures are shown in Fig. (**2**).

**Fig. (2):**
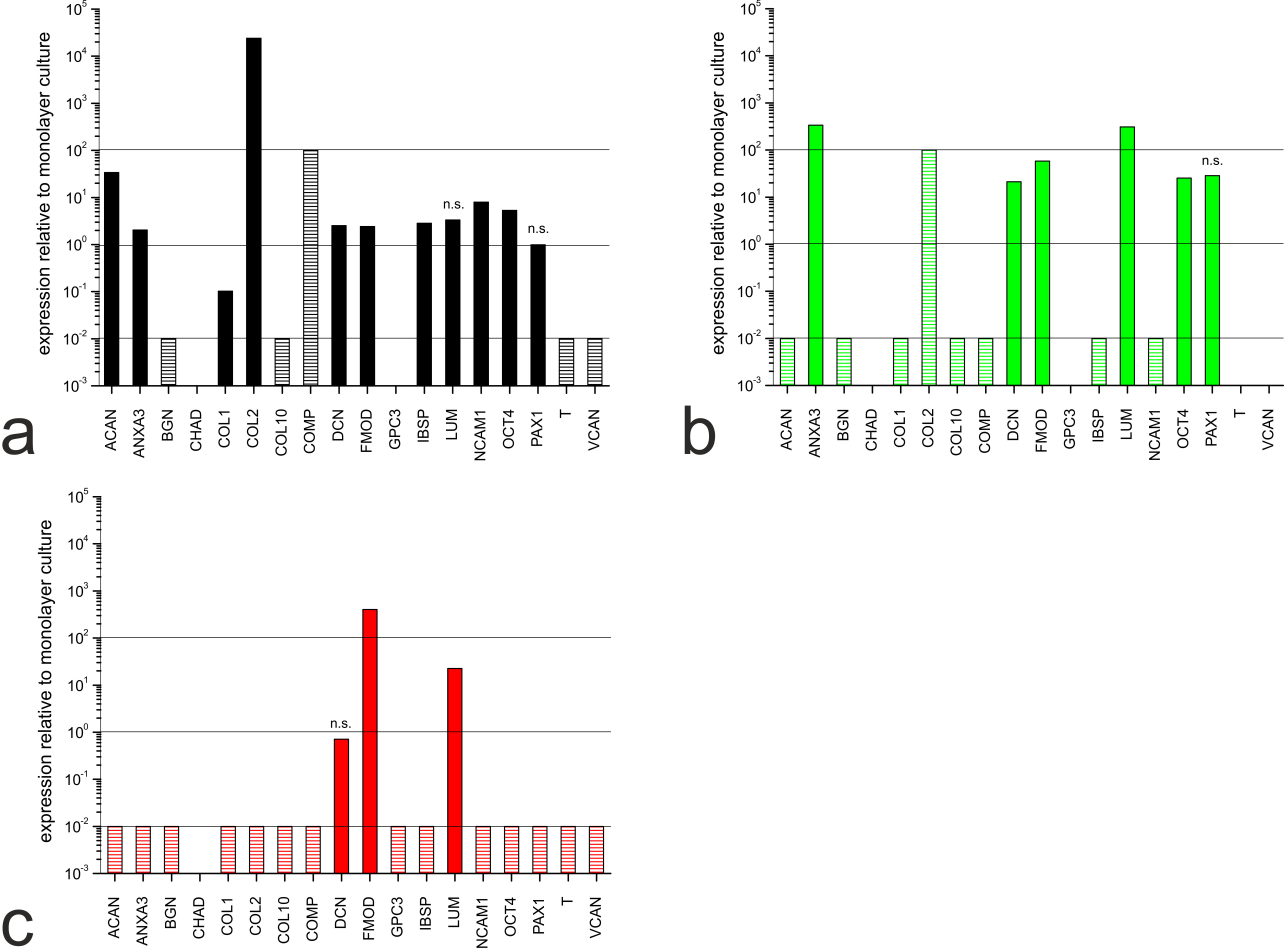
Gene expression profiles in (**a**) hMSC-TERT cells after 1 day in hydrogel culture, and (**b**) NP cells and (**c**) chondrocytes after 21 days in hydrogel culture, using EIF4A2 as the reference gene. C_t_ values were normalized to the reference gene and to the particular mRNA expression in the monolayer culture. No C_t_ value was available for some markers and although a precise quantification was not possible in these cases, expression changes due to culture setting are illustrated by dashed bars. Increased expression is indicated by dashed bars set to 10^2^ (no expression in monolayer but expression detected in hydrogel culture). Decreased expression is indicated by dashed bars set to 10^-2^ (expression detected in monolayer but not in hydrogel culture). Empty spaces indicate no PCR products in the monolayer or hydrogel culture. All calculated relative expression levels were significant (p < 0.05) except those marked as n. s. (= not significant).

Although the hMSC-TERT cells did not undergo differentiation, the gene expression profile had already changed after 1 day in hydrogel culture (Fig. **2a**). Nine markers were upregulated, including those encoding ECM proteins such as collagen type II (COL2) and aggrecan (ACAN) and cell adhesion proteins such as neural cell adhesion molecule 1 (NCAM1). Five markers were downregulated, including those encoding ECM proteins such as collagen type I (COL1), biglycan (BGN) and collagen type X (COLX).

In NP cells, the expression profile had changed after 21 days in hydrogel culture (Fig. **2b**) indicating the differences between dedifferentiated and redifferentiated cells. Six markers were upregulated, including those encoding annexin A3 (ANXA3), which regulates cellular growth, and the proteoglycans lumican (LUM) and fibromodulin (FMOD). The gene encoding collagen type II (COL2) was upregulated but the induction ratio could not be determined because *COL2* was not expressed in the monolayer culture. Seven markers were downregulated, including *ACAN*, *BGN* and *COL1*.

In chondrocytes, 14 of the 18 markers we analysed were downregulated after 21 days in hydrogel culture (Fig. **2c**). This was unexpected, because collagen type I (COL1) is a known marker of chondrocyte redifferentiation and the gene should be upregulated. The genes encoding FMOD and LUM were upregulated, whereas the decorin gene (*DCN*) was not significantly modulated.

### 3.3 Gene Expression Profiles: Comparison of Undifferentiated hMSC-TERT Cells to Dedifferentiated and Redifferentiated NP Cells and Chondrocytes

**Fig. (3):**
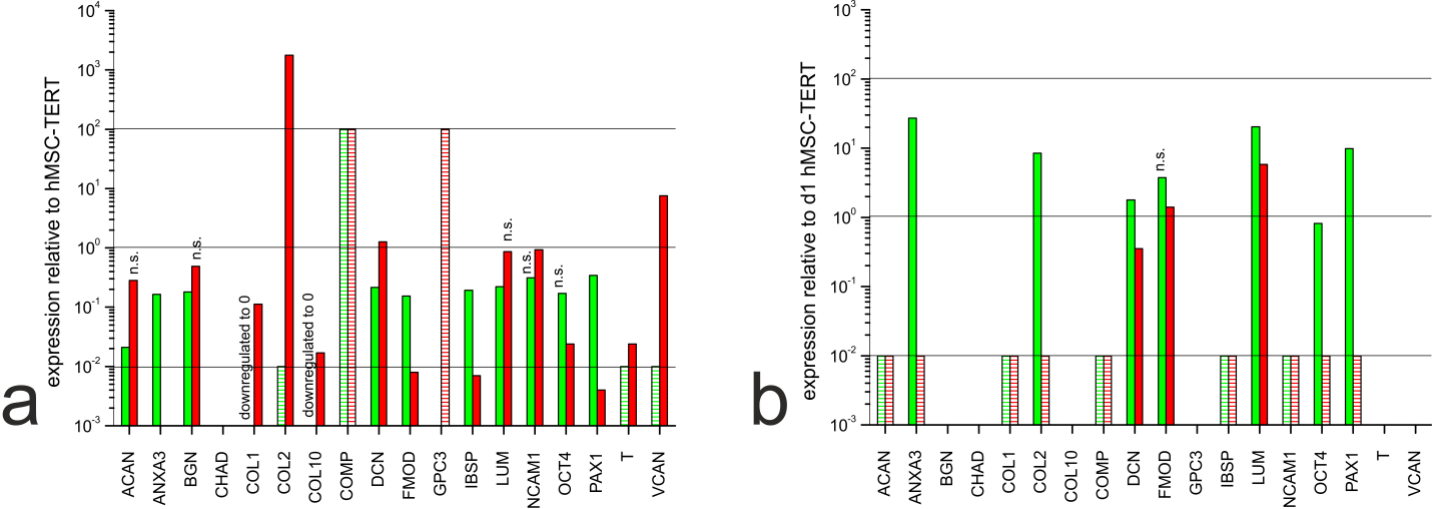
Gene expression profiles of in human NP cells (green) and chondrocytes (red) (**a**) in monolayer culture and (**b**) after 21 days in hydrogel culture, using EIF4A2 as the reference gene. C_t_ values were normalized to the reference gene and to the particular mRNA expression in the hMSC-TERT cells. No C_t_ value was available for some markers and although a precise quantification was not possible in these cases, expression differences among cell types are illustrated by dashed bars. Increased expression is indicated by dashed bars set to 10^2^ (no expression in hMSC-TERT cells but expression detected in the other cells). Decreased expression is indicated by dashed bars set to 10^-2^ (expression detected in hMSC-TERT cells but not in the other cells). Empty spaces indicate no PCR products in any of the cells. All calculated relative expression levels were significant (p < 0.05) except those marked as n. s. (= not significant).

In the monolayer cultures, most of the ECM markers we detected were expressed at similar levels in all three cell types, or expressed more strongly in the hMSC-TERT cells than the dedifferentiated NP cells and chondrocytes (Fig. **3a**). Exceptionally, the genes encoding collagen type II, versican and (to a lesser extent) decorin were expressed more strongly in chondrocytes than in hMSC-TERT cells. In the hydrogel cultures, most markers were expressed more strongly in the hMSC-TERT cells (*e.g.* *ACAN*, *COL1*) and cartilage oligomeric matrix protein (*COMP*) than in the redifferentiated NP cells and chondrocytes (Fig. **3b**). When comparing the two types of redifferentiated cells, we observed the induction of *ANXA3*, *COL2*, *OCT4* and *PAX1* in NP cells compared to chondrocytes, although only *ANXA3*, *COL2* and *PAX1* were suitable as NP markers because *OCT4* was expressed at similar levels in the undifferentiated hMSC-TERT cells.

### 3.4 Raman Spectra: Comparison of Cells in Monolayers and Hydrogels

**Fig. (4):**
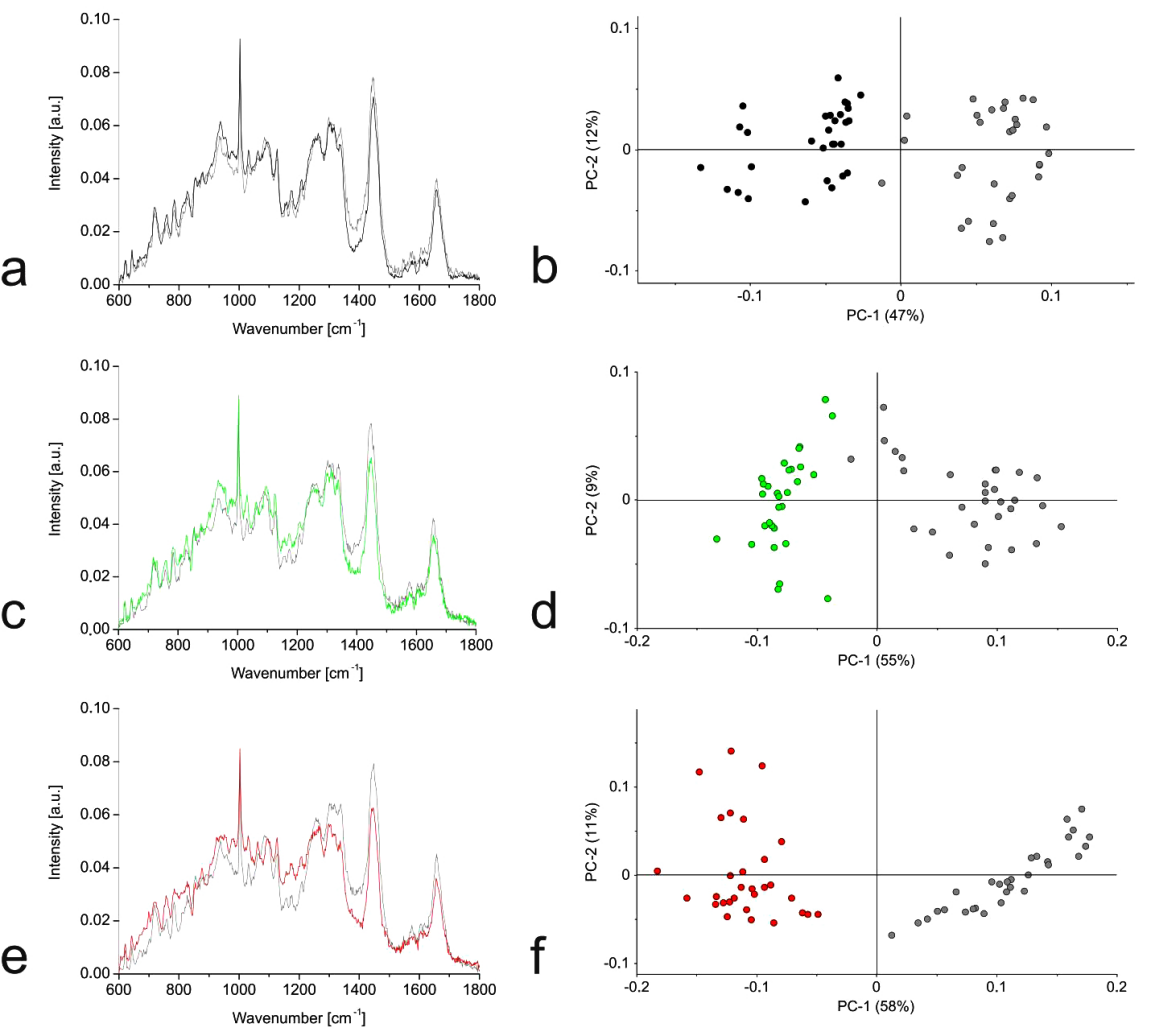
Comparison of cells in monolayer and hydrogel cultures by Raman spectroscopy: hMSC-TERT **(a, b)**, NP cells **(c, d)** and chondrocytes **(e, f)**. Raman spectra from 30 single cells per culture condition were pre-processed, averaged and compared by PCA. **(a, c, e)** Mean Raman spectra of cells in monolayer culture (grey) and (**a**) hMSC-TERT cells (black) after 1 day in hydrogel culture, (**c**) NP cells (green) after 21 days in hydrogel culture, and (**e**) chondrocytes (red) after 21 days in hydrogel culture. **(b, d, f)** PCA score plots based on first (PC1) and second (PC2) principal components. (b) PC1 and PC2 account for 59% of the observed variance between the Raman spectra for hMSC-TERT cells under the two culture conditions. (**d**) PC1 and PC2 account for 64% of the observed variance between the Raman spectra for human NP cells under the two culture conditions. (**f**) PC1 and PC2 account for 69% of the observed variance between the Raman spectra for human chondrocytes under these two culture conditions.

Raman spectra were prepared for the three cell types growing in monolayers and hydrogel cultures. We observed the greatest Raman shifts away from the mean in the chondrocytes and smaller differences for the hMSC-TERT and NP cells (Figs. **4a**, **4c**, **4e**). PCA clearly separated the hydrogel cultures from the monolayer cells (Figs. **4b**, **4d**, **4f**).

### 3.5 Raman Spectra: Comparison of Undifferentiated hMSC-TERT Cells to Dedifferentiated and Redifferentiated NP Cells and Chondrocytes

**Fig. (5):**
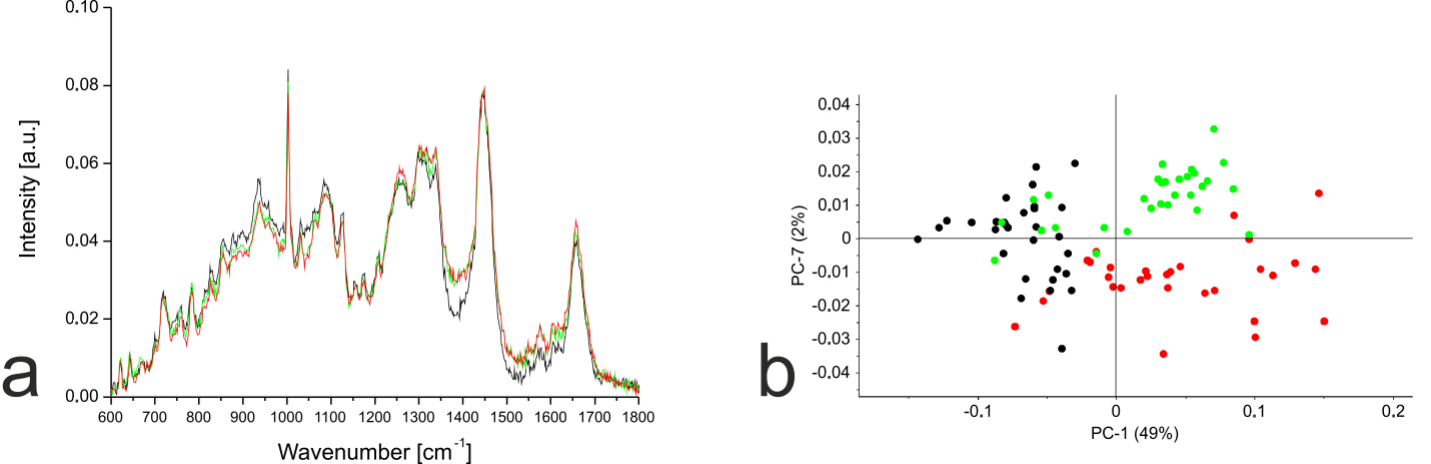
**(a)** Mean Raman spectra for hMSC-TERT cells (black), NP cells (green) and chondrocytes (red) in monolayer culture. Raman spectra from 30 single cells per cell type were pre-processed, averaged and compared by PCA. **(b)** PCA plot comparing hMSC-TERT cells (black), NP cells (green) and chondrocytes (red). Score plot based on first (PC1) and seventh (PC7) principal components. PC1 and PC7 account for 51% of the variance between the Raman spectra for these three cell types.

PCA based on the Raman spectra of the monolayer cells revealed that undifferentiated hMSC-TERT cells were most clearly separated from the other cells along PC1, whereas the dedifferentiated NP cells and chondrocytes were most clearly separated along PC7 (Fig. **5**). Further PCA was carried out using the distinct wavenumbers or wavenumber ranges reported in the PC1 and PC7 loading plots. Detailed analysis of PC1 revealed that the separation between hMSC-TERT cells and the dedifferentiated cells mainly reflected spectral differences in the range 1370-1390. Furthermore, the correlated peak direction in the PC1 loading plot and the assignment of the three cell types in the associated score plot both revealed more biochemical input in the dedifferentiated cells than the hMSC-TERT cells within this range. In the case of PC7, no single wavenumber or wavenumber range contributed mainly to the separation of the dedifferentiated NP cells and chondrocytes.

**Fig. (6):**
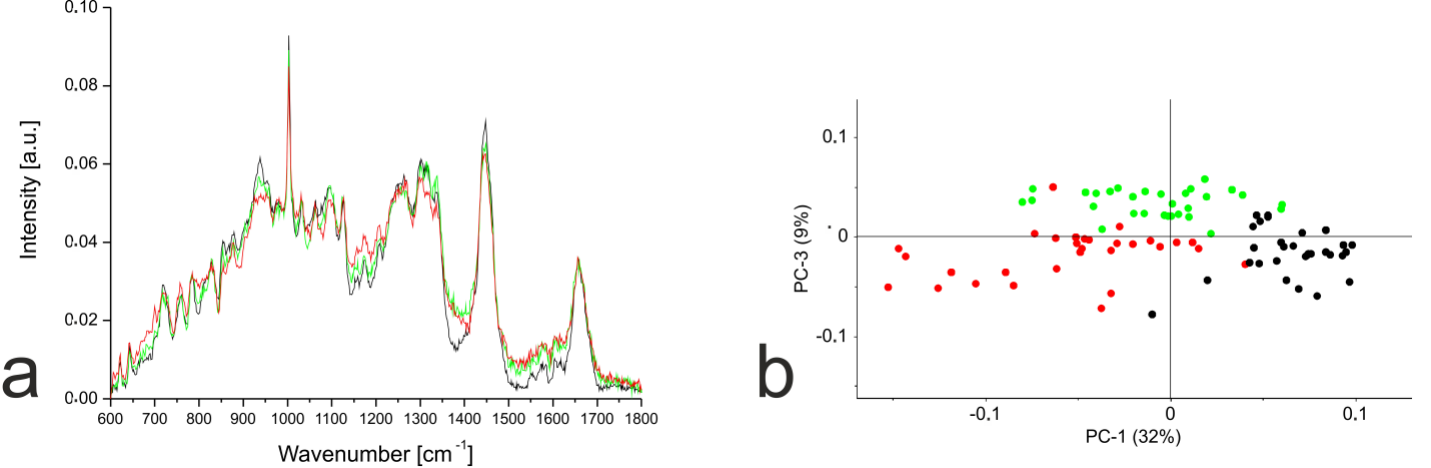
**(a)** Mean Raman spectra for hMSC-TERT cells (black), NP cells (green) and chondrocytes (red) in hydrogel culture (hMSC-TERT after 1 day, others after 21 days). Raman spectra from 30 single cells per cell type were pre-processed, averaged and compared by PCA. **(b)** PCA plot comparing hMSC-TERT cells (black), NP cells (green) and chondrocytes (red). Score plot based on first (PC1) and third (PC3) principal components. PC1 and PC3 account for 41% of the observed variance between the Raman spectra for these three cell types.

In contrast to the monolayer cells, PCA based on the Raman spectra of the hydrogel cultures revealed much clearer separation. The hMSC-TERT cells were most clearly separated from the others along PC1, whereas the redifferentiated NP cells and chondrocytes were separated by PC3 (Fig. **6**). Detailed analysis of PC1 revealed that the separation of hMSC-TERT cells from the redifferentiated NP cells and chondrocytes mainly reflected spectral differences in the wavenumber range 1368–1383, with a peak in the loading plot at 1375. Furthermore, the correlated peak direction in the PC1 loading plot and the assignment of the three cell types in the associated score plot both revealed more biochemical input in the redifferentiated cells than the hMSC-TERT cells within this range. Detailed analysis of PC3 revealed that the separation of the redifferentiated NP cells and chondrocytes mainly reflected spectral differences in the wavenumber range 1329-1345, with peak at 1338. The correlated peak direction in the PC3 loading plot and the assignment of both cell types in the associated score plot both revealed more biochemical input in the NP cells than chondrocytes within this range.

## 4 DISCUSSION

We compared human NP cells, chondrocytes and hMSC-TERT cells in order to find markers that are expressed solely in redifferentiated NP cells to a significantly greater extent in these cells compared to their dedifferentiated counterparts, undifferentiated hMSCs and chondrocytes. Our data offer a clinically relevant starting point to establish markers that can be used to confirm the differentiation of hMSCs into the NP cell lineage. We identified the mRNAs for annexin A3 (*ANXA3*), collagen type II (*COL2*) and paired box 1 (*PAX1*) as the most promising markers based on their preferential strong expression in redifferentiated NP cells.

ANXA3 is a calcium-dependent phospholipid-binding protein that influences membrane organization and traffic [36]. Significantly higher amounts of *ANXA3* mRNA are found in rat NP cells compared to AF and AC tissues [16]. However, the analysis of human cDNA microarrays revealed a slightly lower amount of *ANXA3* mRNA in the NP compared to the AC (NP/AC ratio = –1.08) suggesting that *ANXA3* may be unsuitable as a human NP cell marker [13]. We were unable to calculate the precise *ANXA3* mRNA NP/AC because the transcript was not detected in chondrocytes, but the abundance of this message in NP cells coupled with its negligible accumulation in chondrocytes suggests that the ratio is very high, contradicting the earlier study [13]. Healthy human NP tissue is scarce (most is obtained from post-mortem examinations) so we decided to test commercially available human NP cells. These are derived from a foetal donor at 20 weeks gestation, indicating that the developmental phenotype of the cells is likely to be closer to the notochord than the mature NP. Although the mean age of the NP donors was not reported in the earlier study, there is probably a large age difference compared to our donor material, and this is the most likely explanation for the contradictory expression profiles. Members of the annexin family regulate cell growth and signal transduction which may explain the higher expression levels in younger NP cells. These issues can be addressed by characterizing the expression of *ANXA3* mRNA in NP cells taken from older donors.

Collagen type II is a major protein component of the ECM secreted by NP cells, but because it is also present in the ECM of the AC, it cannot be used as the sole marker for NP redifferentiation. Even so, our data clearly show that COL2 is expressed significantly more strongly in redifferentiated NP cells compared to hMSC-TERT cells, so it can be used to indicate the change from a stem cell character to the NP phenotype, meaning successful differentiation.

PAX1 is a transcription factor that regulates vertebral segmentation during embryogenesis [37]. *PAX1* mRNA was previously shown to be more abundant in human NP cells than AC cells, and to be present at negligible levels in hMSCs followed by a significant increase following 14 days of chondrogenic differentiation [13]. Although *PAX1* expression profiles were not directly compared in NP cells, AC cells and hMSCs, the data indicate that minimal *PAX1* expression in undifferentiated hMSCs switches to stronger expression in redifferentiated NP and AC cells. Our direct quantitative comparison of all three relevant cell types revealed higher PAX1 expression in NP cells compared to both chondrocytes and hMSCs, therefore confirming the suitability of *PAX1* mRNA as a NP marker during regeneration.

Raman spectra have previously been reported for hMSCs and chondrocytes [28]. Such spectra are usually displayed as specific intensities dedicated to corresponding single Raman shifts (cm^-1^) measured in arbitrary units, but the data can be processed in different ways and are not always suitable for direct comparison. Although we cannot compare our results quantitatively with these previous studies, the mean Raman spectra and corresponding PCA data can be interpreted and discussed in the context of earlier reports of Raman shifts corresponding to biological and/or biochemical assignments to different types of tissue [38, 39] including cartilage [31, 40].

The Raman spectra from our monolayer cells revealed that hMSC-TERT cells could be distinguished from dedifferentiated NP cells and chondrocytes because the latter cells produce more intense Raman shifts in the range 1370-1390 cm^-1^. As previously reported [40], the 1380 cm^-1^ shift can be assigned to glycosaminoglycans (GAGs), indicating that chondrocytes and NP cells have a higher GAG content than hMSCs, which is expected given that GAGs are major ECM components of the AC [41] and NP [12].

 Although monolayers of human NP cells and chondrocytes could be separated along PC7, the loading plot revealed no wavenumber range contributing primarily to this separation. Dedifferentiated NP cells and chondrocytes therefore generate different Raman spectra, indicating different overall biochemical composition, but there does not appear to be a major molecule or cellular component responsible for this difference.

Therapeutically more relevant is the non-invasive distinction of the three cell types in 3D culture. Here, redifferentiated NP cells and chondrocytes could be separated from undifferentiated hMSC-TERT cells due to more intense Raman shifts at 1375 cm^-1^, again representing GAGs [40]. Detailed analysis of PC3, which separated the two redifferentiated cell types, revealed that most of the separation is contributed by Raman shifts close to 1338 cm^-1^, which again represents GAGs [40], and that the NP cells contribute a greater biochemical input and therefore have a higher GAG content.

## CONCLUSION

We have demonstrated that hMSCs, NP cells and chondrocytes produce Raman spectra that are not only cell type-specific but also differentiation status-dependent, allowing this non-invasive analytical method to be used to distinguish among different cell types during the regeneration of NP tissue.

 We have also shown that *ANXA3*, *COL2* and *PAX1* mRNAs offer promising markers for human native NP cells, because all three are expressed at significantly higher levels in redifferentiated human NP cells compared to dedifferentiated NP cells, chondrocytes and undifferentiated hMSCs. We therefore recommend *ANXA3*, *COL2* and *PAX1* as ideal markers for human NP cells when it is necessary to distinguish them from chondrocytes and/or hMSCs. These *in vitro* results should be evaluated in more detail to determine whether the markers can also be used *in vivo*,

## CONSENT FOR PUBLICATION

Not applicable.

## Figures and Tables

**Table 1:** qPCR primer sequences.

**Gene Symbol**	**Accession** **Number**	**Direction** **(5´→ 3´)**	**Sequence**	**Tm (°C)**	**Product** **Length (bp)**
*EIF4A2*	not published by manufacturer Primerdesign				113
*ACAN*	NM_001135.3	F	AGGGGACTTCCGCTGGTCAGATG	66.0	197
		R	TGCGTTTGTAGGTGGTGGCTGTG	64.2	
*ANXA3*	NM_005139.2	F	TGGGTTGGACACCGAGGAACAGT	64.2	148
		R	GCCGCTGTGCATTTGACCTCTCA	64.2	
*BGN*	NM_001711.4	F	CACCAAAGTGGGTGTCAACG	59.6	70
		R	GATGCCGTTGTAGTAGGCCC	60.5	
*CHAD*	NM_001267.2	F	TCTCAGATGGTGCCTTCCTGGGTG	66.1	128
		R	GGGGTTATTGGTAAGGGCGAGGGT	66.1	
*COL1A1*	NM_000088.3	F	GAGTGGTGATCGTGGTGAGACTGGT	66.3	139
		R	CTTTATGCCTCTGTCGCCCTGTTCG	66.3	
*COL2A1*	NM_033150.2	F	GAAGAACTGGTGGAGCAGCAAGAGC	66.3	147
		R	GACAGCAGGCGTAGGAAGGTCATCT	66.3	
*COL10A1*	NM_000493.3	F	ATGGGATATGGTGCTCCTGGTCGTC	66.3	133
		R	CTTTGATGCCTGGCTGTCCTGGAAC	66.3	
*COMP*	NM_000095.2	F	GCAGGTCAGGGAGATCACGTTCCT	66.1	78
		R	GTGCGTACTGACTGCTGCATCCC	66.0	
*DCN*	NM_001920.3	F	TTCCTGATGACCGCGACTTC	60.1	70
		R	CGAAGATGGCATTGACAGCG	60.0	
*FMOD*	NM_002023.3	F	GGCCTTGTACCTCCAACACA	59.9	78
		R	TCCAGCAAGATCAGTGACCG	59.8	
*GPC3*	NM_001164617.1	F	GCAGGTGTGGTGGAGATTGACAAGT	64.6	184
		R	TCTCAGTTTCAGTGGTGGTCAGCTT	63.0	
*IBSP*	NM_004967.3	F	GGGCACCTCGAAGACAACAACCTC	66.1	120
		R	TCCCCCTCGTATTCAACGGTGGTG	66.1	
*LUM*	NM_002345.3	F	GCAAGATCCTGGGGCCATTA	59.8	87
		R	CCGGTGGAAGACTGGTTTCT	59.6	
*NCAM1*	NM_000615.6	F	TGTGTCGTCGCTGACCCTGAAGAG	66.1	172
		R	GTTCACCTGGTTCCCCTCCCAAGT	66.1	
*OCT4*	NM_002701.5	F	ATTCAGCCAAACGACCATCTGCCG	64.4	75
		R	AAGGGCCGCAGCTTACACATGTTC	64.4	
*PAX1*	NM_006192.4	F	TTCAAGCATCCCAGCCGAGAAGGA	64.4	79
		R	AGTCCGTGTAAGCTACTGAGGGCG	66.1	
*T*	AJ001699.1	F	ACCCTGTGTCCACCTGCAAATCCT	64.4	133
		R	GATGAGCATAGGGGCTGGGGTAGG	67.8	
*VCAN*	NM_004385.4	F	GTGGAGGTGGTCTACTTGGGGTGAG	67.9	156
		R	AACTGGGTGATGCAGTTTCTGCGAG	64.6	
